# DNA methylation age-acceleration is associated with disease duration and age at onset in *C9orf72* patients

**DOI:** 10.1007/s00401-017-1713-y

**Published:** 2017-04-24

**Authors:** Ming Zhang, Maria Carmela Tartaglia, Danielle Moreno, Christine Sato, Paul McKeever, Anna Weichert, Julia Keith, Janice Robertson, Lorne Zinman, Ekaterina Rogaeva

**Affiliations:** 1 Tanz Centre for Research in Neurodegenerative Diseases, University of Toronto, 60 Leonard Ave., Toronto, ON M5T 2S8 Canada; 2 Division of Neurology, Department of Medicine, University of Toronto, 1 King’s College Circle, Toronto, ON M5S 1A8 Canada; 3 Krembil Neuroscience Center, University Health Network Memory Clinic, Toronto Western Hospital, 399 Bathurst St., Toronto, ON M5T 2S8 Canada; 4 Department of Laboratory Medicine and Pathobiology, 27 King’s College Circle, Toronto, ON M5S 1A1 Canada; 5 Sunnybrook Health Sciences Centre, 2075 Bayview Ave., Toronto, ON M4N 3M5 Canada

**Keywords:** ALS, FTD, *C9orf72*, DNA methylation age

## Abstract

The repeat expansion in *C9orf72* is the most common cause of amyotrophic lateral sclerosis and frontotemporal dementia. *C9orf72* patients present with a wide range in disease duration and age of onset. The strongest risk factor for both syndromes is aging, which was linked to DNA methylation (DNAm) age based on the cumulative assessment of the methylation levels of 353 CpGs included on the genome-wide 450k BeadChip. DNAm age may reflect biological age better than chronological age. We conducted a genome-wide blood DNA methylation study of 46 unrelated *C9orf72* patients. After correction for multiple testing, none of the CpGs demonstrated association between its methylation level and disease duration or age of onset. However, we detected a significant reverse correlation of DNAm age-acceleration with disease duration and age of onset, suggesting that for every 5-year increase in DNAm age-acceleration there is a 3.2-year earlier age of onset and 1.5-year shorter disease duration. The significant correlations remain after adjusting for gender, *TMEM106B* genotypes, disease phenotype and *C9orf72* 5′CpG island methylation status. A similar trend was observed for the blood DNA of affected members of an extended *C9orf72* family; and tissues from the central nervous system of *C9orf72* autopsy cases. For instance, regression analysis suggested that a 5-year increase in DNAm age-acceleration is linked to an earlier age of onset by 4.7 or 5.5 years for frontal cortex or spinal cord, respectively. Blood DNAm age may be a useful biomarker for biological age, because blood DNAm age-acceleration was similar to all investigated brain tissues, except for cerebellum that ages more slowly. In conclusion, DNA methylation analysis of *C9orf72* patients revealed that increased DNAm age-acceleration is associated with a more severe disease phenotype with a shorter disease duration and earlier age of onset.

**Electronic supplementary material:**

The online version of this article (doi:10.1007/s00401-017-1713-y) contains supplementary material, which is available to authorized users.

## Introduction

Most patients with amyotrophic lateral sclerosis (ALS) and frontotemporal dementia (FTD) have a sporadic form of the disease suggesting the contribution of several small-effect genetic and environmental risk factors that could be linked to DNA methylation changes [1]. Even Mendelian ALS and FTD genes show pleiotropy, in which the same mutation can result in different phenotypes with variable severity [9]. For instance, the G_4_C_2_-repeat expansion in *C9orf72* is the most common known cause of both ALS and FTD [7, 26], accounting for ~37% familial and ~7% sporadic patients [24]. In addition to variable syndromes, the phenotypic heterogeneity in *C9orf72* patients includes a wide range in disease duration (0.5–22 years) and age of onset (27–74 years) [5, 21]. However, knowledge about disease modifiers is limited. Currently, only variations in *ATXN2* and *TMEM106B* have been suggested as genetic modifiers in *C9orf72* carriers. Intermediate *ATXN2* alleles (27–33 CAG-repeats) were reported as modifiers in *C9orf72* carriers, rendering susceptibility to ALS [29]; and homozygosity for the minor allele (G) of rs3173615 in *TMEM106B* was reported to protect against developing FTD in *C9orf72* patients [30], while the major allele (A) of rs1990622 in *TMEM106B* was associated with a later age of onset in *C9orf72* FTD patients [8].

The strongest risk factor for neurodegenerative diseases (e.g., ALS and FTD) is aging, which was linked to the epigenetic clock called DNA methylation (DNAm) age that is an accurate predictor of chronological age across different tissues [10]. DNAm age is based on the cumulative assessment of the methylation levels of 353 CpGs included on a genome-wide array (methylation levels of 193 CpGs increase with age, while methylation levels of 160 CpGs decrease with age). Age-related CpGs are mainly mapped to genes involved in cell death, survival and development. Increased DNAm age-acceleration (DNAm age minus chronological age) is associated with several disorders, including Parkinson’s disease [14], Huntington’s disease [12] and Down syndrome [11]. Therefore, DNAm age may reflect biological age better than chronological age.

Aberrant DNA methylation was reported to be involved in several neurodegenerative diseases, including Alzheimer’s disease [6, 37], Huntington’s disease [31], dementia with Lewy bodies and Parkinson’s disease [28]. DNA hypermethylation was found at the CpG island 5′ of the G_4_C_2_-repeat in *C9orf72* patients [18, 36], which was associated with longer disease duration and later age of death in *C9orf72* FTD patients [27]. Furthermore, we recently detected DNA hypermethylation of the G_4_C_2_-repeat itself in carriers of a large expansion in *C9orf72* (currently, a 90-repeat allele is the longest expansion reported to be free from methylation) [35]. The role of environmental/epigenetic factors in ALS is supported by studies of monozygotic (MZ) twins discordant for the disease [2]. For instance, we reported the *C9orf72* expansion in a MZ twin-pair discordant for ALS for 7 years [34]. Their identical genetic background, similar repeat size and degree of methylation at the *C9orf72* locus [36] indicate the contribution of environmental factors. Indeed, only the affected twin had a prominent history of smoking and head injury [34], which may influence DNA methylation [17]. Notably, the difference in DNAm age suggested that the affected twin had aged faster than the asymptomatic twin; and a similar trend was detected in another pair of MZ twins carrying the *SOD1* and *ARHGEF28* mutations, who were ALS-discordant for 17 years [38].

However, it remains unknown if genome-wide DNA methylation changes contribute to the diversity of *C9orf72* phenotypes. Hence, we conducted a genome-wide DNA methylation study in a Canadian cohort of *C9orf72* carriers. Our findings strongly suggest that increased DNAm age-acceleration is linked to shorter disease duration and younger age at onset.

## Materials and methods

### Human samples

Informed consent was obtained from each participant in accordance with the ethics review board. Blood DNA samples were collected from 46 unrelated *C9orf72* patients of Caucasian ethnicity, which were diagnosed in Toronto at either the ALS Clinic in the Sunnybrook Health Sciences Centre (31 ALS and 6 ALS-FTD patients) or the University Health Network Memory clinic (9 FTD patients) using established clinical criteria [4, 25]. The characteristics of the dataset are presented in Table 1. In addition, a family based study of DNAm age-acceleration was conducted for a British-Canadian *C9orf72* ALS family (PED25), for which genetic and clinical data were published previously [33]. Briefly, a 70-repeat allele from the father (unaffected by ALS or FTD at age 90) expanded during parent-offspring transmission and started the first generation affected by ALS with four of five children carrying a large expansion (~1750 repeats).

**Table 1:** Clinical information of the *C9orf72* patients included in blood DNA methylation analyses

	Entire patient group	ALS patients	ALS-FTD patients	FTD patients
Number of samples	46	31	6	9
Familial cases	74%	74%	67%	78%
Males	50%	45%	100%	44%
Age of onset (years, mean ± SEM)	58.8 ± 1.2	60.1 ± 1.5	55.5 ± 3.0	56.8 ± 2.6
Disease duration (years, mean ± SEM)	3.7 ± 0.5	2.9 ± 0.4	5.3 ± 2.4	7.0 ± 1.3

We also investigated 46 DNA samples from frozen central nervous system (CNS) tissues (spinal cord, cerebellum, frontal, motor, and temporal cortex) obtained from up to ten unrelated autopsy *C9orf72* cases (Table 2).

**Table 2:** Clinical information of the autopsy *C9orf72* cases included in CNS DNA methylation analyses

	Cerebellum	Spinal cord	Frontal cortex	Motor cortex	Temporal cortex
Number of samples	9	9	10	9	9
Number of ALS samples	6	6	7	6	6
Number of ALS-FTD samples	3	3	3	3	3
Males	56%	67%	60%	56%	56%
Age of onset (years, mean ± SEM)	54.6 ± 1.8	56 ± 2.4	55.8 ± 2.3	54.6 ± 1.8	54.6 ± 1.8
Disease duration (years, mean ± SEM)	2.67 ± 0.24	2.44 ± 0.29	2.56 ± 0.23	2.67 ± 0.24	2.67 ± 0.24

### Genetic analyses

All subjects were genotyped previously by repeat-primed PCR [36] and have typical large expansions based on the methylation of the G_4_C_2_-repeat in *C9orf72* [35], except the sample with a 70-repeat allele from PED25. The entire cohort was also genotyped for the CAG-repeats in *ATXN2* as previously reported [39], and two *TMEM106B* variations by Sanger sequencing using the primers 5′-GCATTGTGTTTGATTGTAGGGG-3′ and 5′-ACTCCAGGACTTATGTGGCC-3′ for rs1990622 and 5′-ACTTGTAAATTTTCTGTGTCCTT-3′ and 5′-CTGTACCCAGCAGAGACACA-3′ for rs3173615. The genotyping results of *TMEM106B* and *ATXN2* can be found in Table S1.

### DNA methylation analyses

DNA was bisulfite converted using the EZ DNA Methylation-Lightning^™^ Kit (Zymo). Genome-wide methylation profiling was performed using the Infinium HumanMethylation 450k BeadChip (Illumina, #WG-314-1003) following the manufacturer’s instructions. The *β* value was used to estimate the methylation level of each CpG site using the ratio of intensities between methylated and unmethylated alleles. *β* values range from 0 (non-methylated) to 1 (completely methylated). The raw data was analyzed using the minfi package in R-project [3]. In brief, the raw data was processed by quantile normalization and CpGs that could be affected by known common variations (minor allele frequencies >1%) were removed from the analysis.

Raw data from the 450k BeadChip was generated using the Illumina GenomeStudio Software (version 2011.1), and then uploaded to the online DNAm age calculator tool (https://dnamage.genetics.ucla.edu), which uses a panel of 353 CpGs to estimate DNAm age based on an elastic net regression model [10]. DNAm age-acceleration was calculated as DNAm age minus chronological age (the date of sample collection minus date of birth).

In addition, we estimated the number of methylated CpG sites at the CpG island 5′ of the G_4_C_2_-repeat for 15 recently collected *C9orf72* subjects using bisulfite sequencing as previously reported [36] (for the rest of the samples in our cohort, methylation data were available from previous studies [32, 36]).

### Statistics

The *F* test was used to estimate the association between the locus-by-locus DNA methylation changes and disease duration or age of onset, as well as evaluate the false discovery rate to generate an adjusted *q* value correcting for multiple comparisons. The Pearson’s correlation analysis was used to estimate the link between DNAm age-acceleration and disease duration or age of onset. Linear regression was used to assess if the correlation fits a linear model. For the blood DNAm age analyses, multivariate linear regression was used to obtain *p* values adjusted for gender, disease phenotype, *TMEM106B* genotypes, methylation status of the 5′CpG island of *C9orf72* and age of onset (when testing for disease duration). The one way ANOVA with Bonferroni post hoc test was used to compare the mean difference between the investigated tissues. A corrected *p* value <0.05 or *q* value <0.05 was accepted as statistically significant. R-project 3.3.1 and SPSS version 20 (IBM) were used for the statistical analysis.

## Results

### Genome-wide CpG methylation analyses

We conducted a genome-wide DNA methylation profiling of blood DNA from 46 unrelated *C9orf72* patients (Table 1) to estimate if a difference in methylation level at any single CpG site on the 450k BeadChip is associated with age of onset or disease duration (age at death minus age of onset). None of the CpGs demonstrated significant association between its methylation level and disease duration or age of onset. The top 20 nominally significant CpGs (*p* < 0.0001, but *q* > 0.05) are listed in Tables S2–S3.

### DNAm age-acceleration using blood DNA

**Fig. 1:**
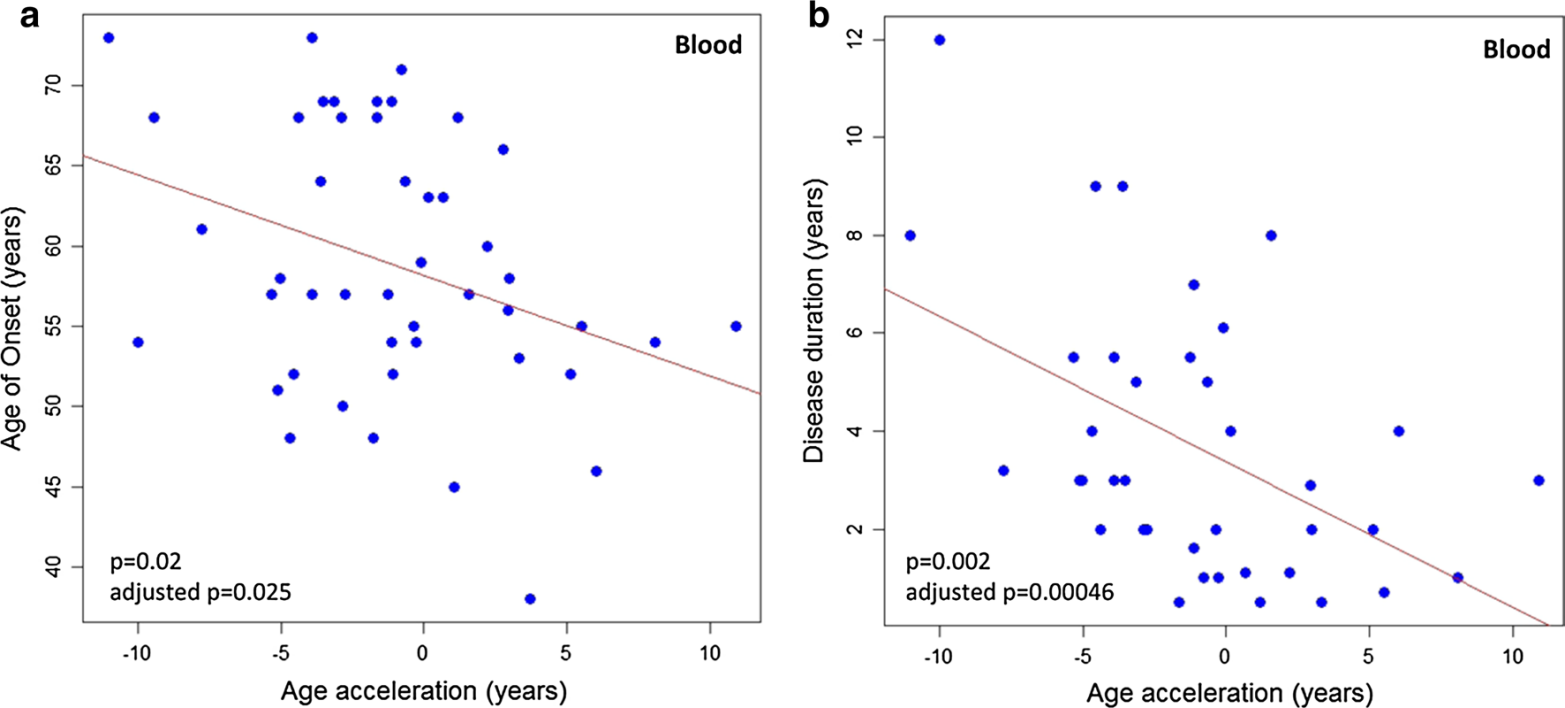
*Scatter plots* demonstrating significant reverse correlation of blood DNAm age-acceleration with disease duration or age of onset among 46 *C9orf72* patients. **a** The correlation between DNAm age-acceleration and age of onset (Pearson correlation coefficient = −0.343; *p* = 0.02; adjusted *p* = 0.025, adjusted beta = −0.334). The *line* represents a fitted linear regression, suggesting age-acceleration is reversely correlated to age of onset (age of onset = 58.2 − 0.63 × age-acceleration). **b** The correlation between DNAm age-acceleration and disease duration (Pearson correlation coefficient = −0.49; *p* = 0.002; adjusted *p* = 0.00046, adjusted beta = −0.519). The *line* represents a fitted linear regression, suggesting the age-acceleration is inversely correlated to disease duration (disease duration = 3.4 − 0.3 × age-acceleration)

Among the 46 unrelated *C9orf72* patients, we detected a significant reverse correlation of blood-based DNAm age-acceleration with age of onset (Pearson correlation coefficient = −0.343; *p* = 0.02; and adjusted *p* = 0.025, adjusted beta = −0.334) (Fig. 1a) and disease duration (Pearson correlation coefficient = −0.49; *p* = 0.002; and adjusted *p* = 0.00046, adjusted beta = −0.519 (Fig. 1b). The regression model suggests that for every 5-year increase in DNAm age-acceleration there is a 3.2-year earlier age of onset (Fig. 1a) and 1.5-year shorter disease duration (Fig. 1b). The observed correlations were unlikely affected by the size of the expansion in *C9orf72*, because all patients carry a large methylated G_4_C_2_-expansion, or by *ATXN2*, because all subjects had a normal number of CAG-repeats (<27). The multivariate linear regression results can be found in Table S4.

**Fig. 2:**
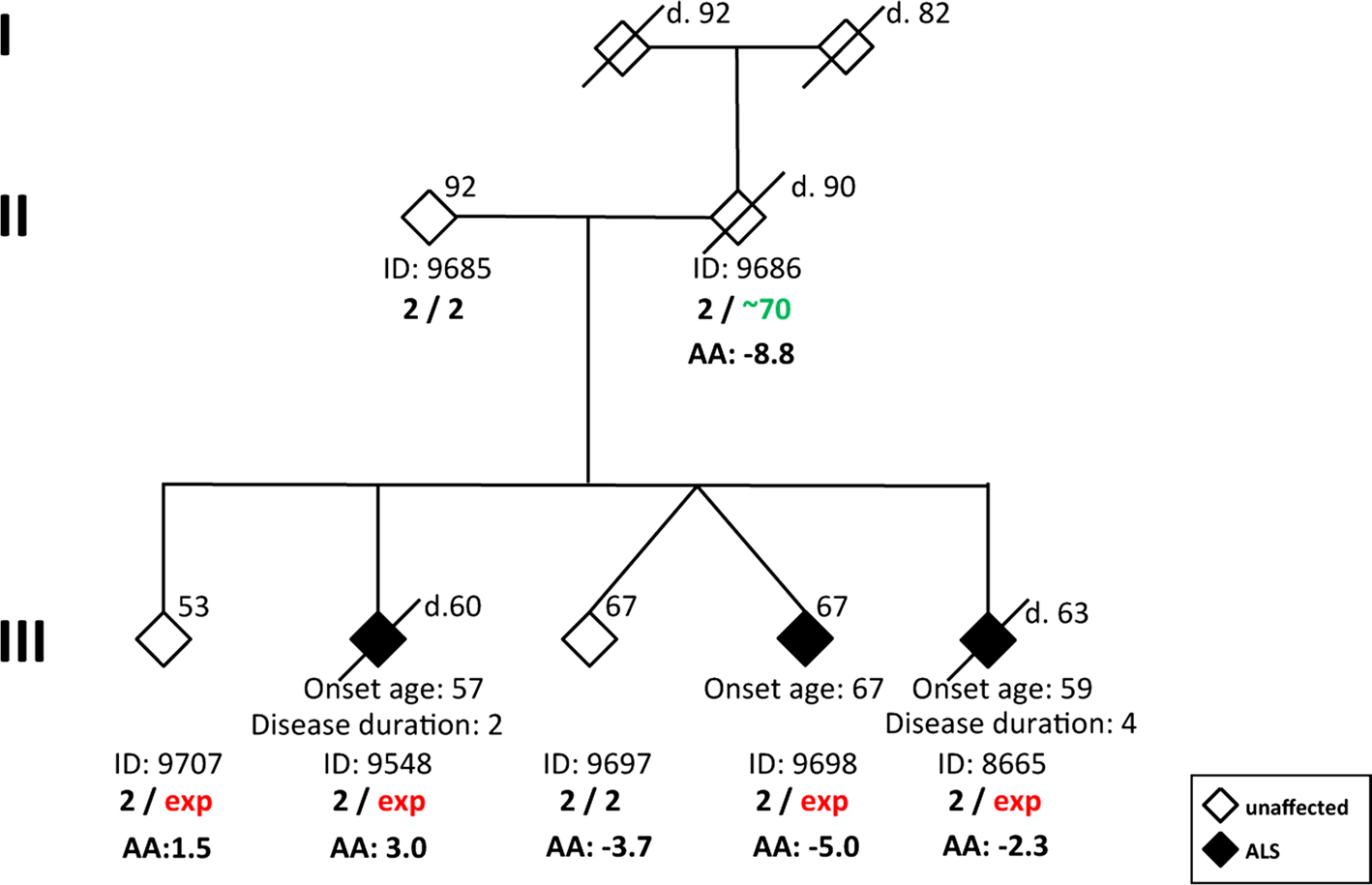
Investigation of DNAm age-acceleration in PED25 family demonstrated that increased DNAm age-acceleration (AA) corresponds to an earlier age at onset and shorter disease duration

Next, we investigated blood DNA samples from five members of a *C9orf72* family (PED25), including four siblings carrying the large expansion with three of them affected by ALS (Fig. 2). In concordance with the results above, we found that an earlier age at onset or shorter disease duration was accompanied by an increase in DNAm age-acceleration. In patient #9548 with an age at onset of 57, age-acceleration = 3.0 years; in patient #8665 with an age at onset of 59, age-acceleration = −2.3 years; and in patient #9698 with age at onset of 67, age-acceleration = −5.0 years. Hence, we observed an 8-year difference in DNAm age-acceleration between the siblings #9548 and #9698 with a disease onset 10 years apart. A similar trend was detected for disease duration in two deceased siblings. Patient #9548 had a 2-year duration with age-acceleration = 3.0 years, while patient #8665 had a 4-year duration with age-acceleration = −2.3 years. Notably, the father #9686 with a pre-mutation (70-repeat allele), who died at age 90 without ALS or FTD symptoms, had the lowest DNAm age-acceleration (−8.8 years). The youngest 53-year-old *C9orf72* carrier #9707 is currently asymptomatic (DNAm age-acceleration = 1.5 years).

### DNAm age-acceleration using CNS tissues

**Fig. 3:**
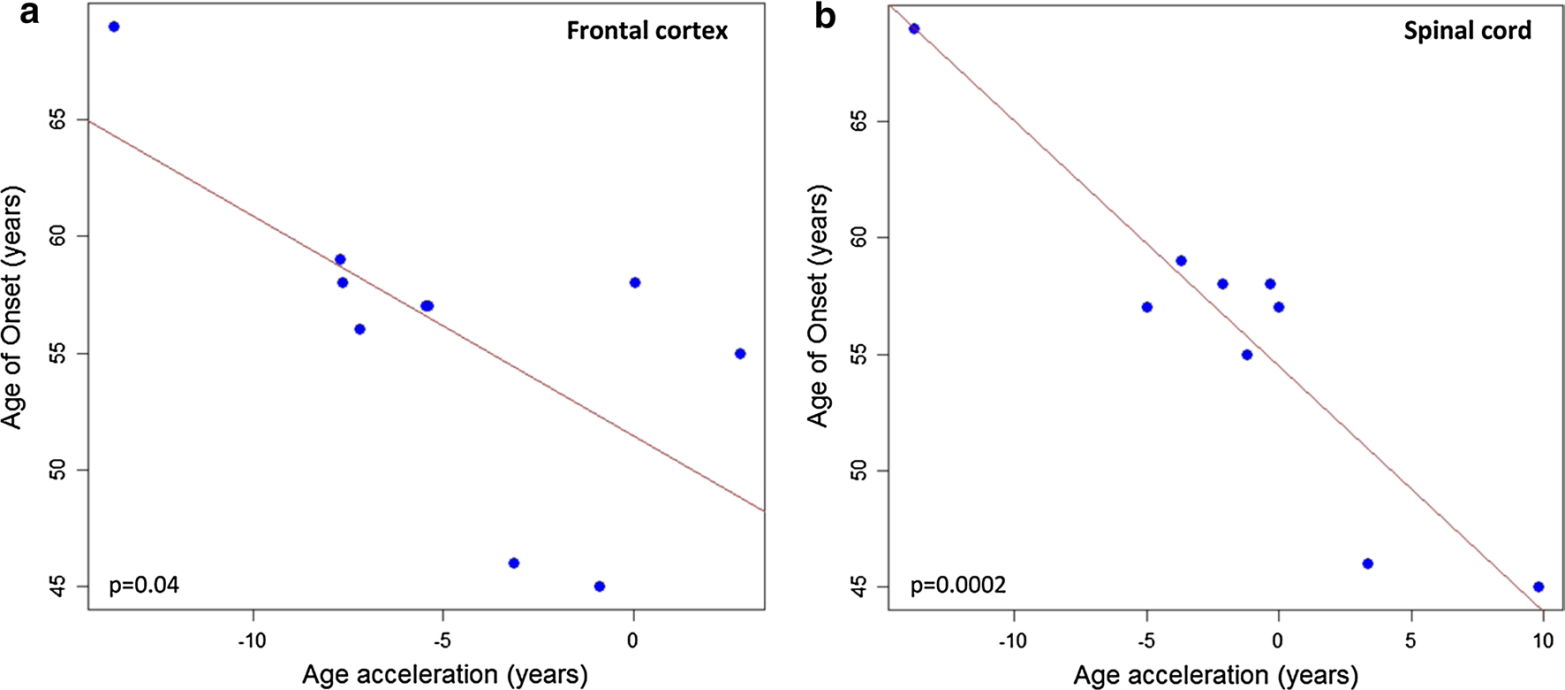
*Scatter plots* demonstrating significant reverse correlation of DNAm age-acceleration in frontal cortex or spinal cord with age of onset in *C9orf72* cases. **a** The correlation between DNAm age-acceleration and age of onset in frontal cortex (Pearson correlation coefficient = −0.657, *p* = 0.04). The *line* represents a fitted linear regression, suggesting that DNAm age-acceleration is inversely correlated to age of onset (age of onset = 51.5 − 0.94 × age-acceleration). **b** The correlation between DNAm age-acceleration and age of onset in spinal cord (Pearson correlation coefficient = −0.935, *p* = 0.0002). The *line* represents a fitted linear regression, suggesting that age-acceleration is inversely correlated to age of onset (age of onset = 54.5 − 1.1 × age-acceleration)

**Fig. 4:**
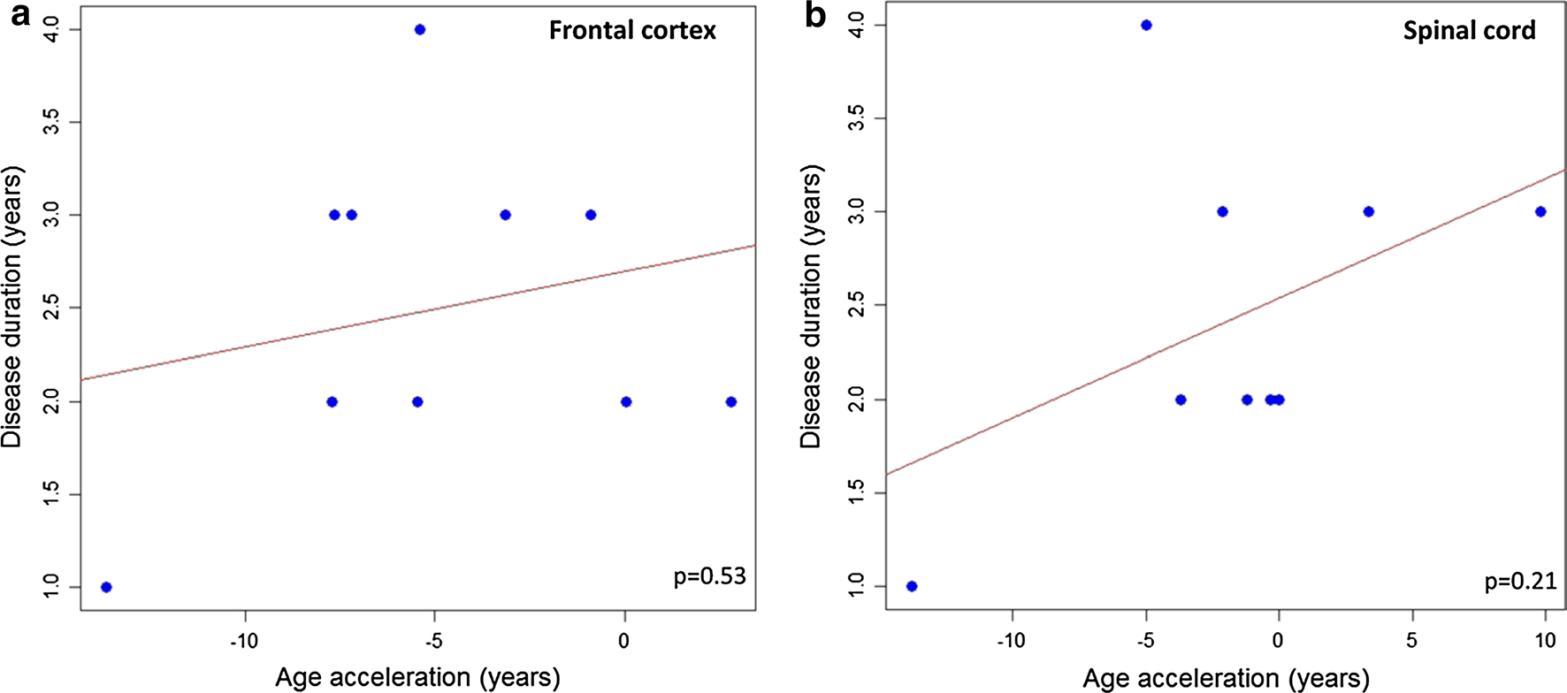
In the *C9orf72* autopsy cases, DNAm age-acceleration is not associated with disease duration in frontal cortex (**a**) or spinal cord (**b**) (*p* > 0.05)

**Fig. 5:**
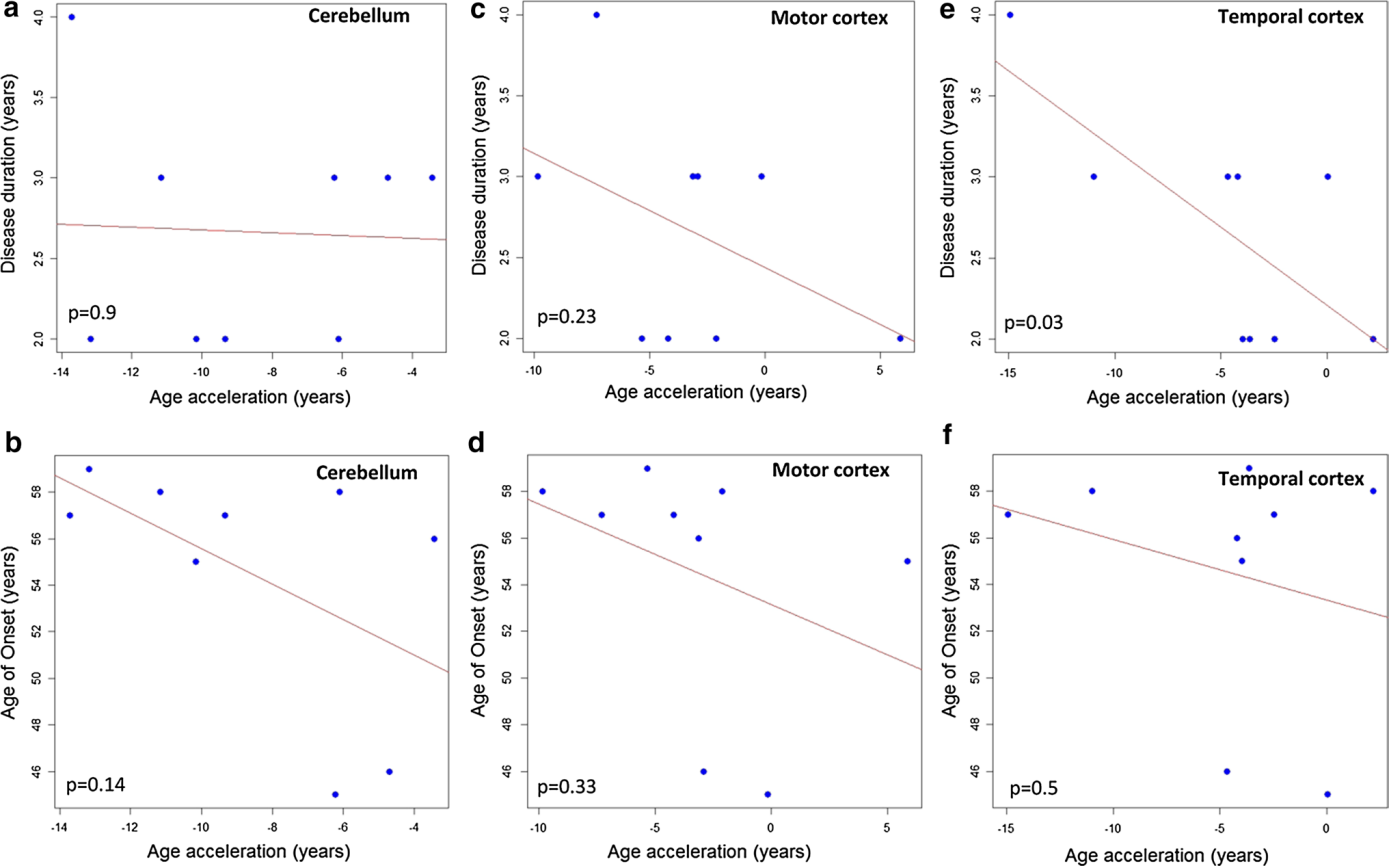
There was no significant correlation between DNAm age-acceleration and disease duration or age at onset using DNA from cerebellum or motor cortex of the *C9orf72* cases (**a–d**). In temporal cortex, DNAm age-acceleration significantly correlated with disease duration (Pearson correlation coefficient = −0.715, *p* = 0.03) (**e**), but not age at onset (**f**)

In a pilot study of moderate cohort, we assessed the correlation between DNAm age-acceleration and disease duration or age of onset in five disease-relevant CNS tissues of *C9orf72* autopsy cases diagnosed with ALS or ALS-FTD (Table 2). Increased DNAm age-acceleration correlated with an earlier age of onset using DNA from frontal cortex (Pearson correlation coefficient = −0.657, *p* = 0.04) (Fig. 3a) and spinal cord (Pearson correlation coefficient = −0.935, *p* = 0.0002) (Fig. 3b), but not with disease duration (Fig. 4a, b). Regression analyses showed that the correlations fit a linear model, suggesting that a 5-year increase in DNAm age-acceleration based on DNA from frontal cortex or spinal cord is linked to a 4.7- or 5.5-year earlier age of onset, respectively (Fig. 3a, b). We did not observe significant results using DNA from cerebellum or motor cortex (Fig. 5a–d). However, in temporal cortex, DNAm age-acceleration fits a linear model and correlates with disease duration (Pearson correlation coefficient = −0.715, *p* = 0.03) (Fig. 5e).

**Fig. 6:**
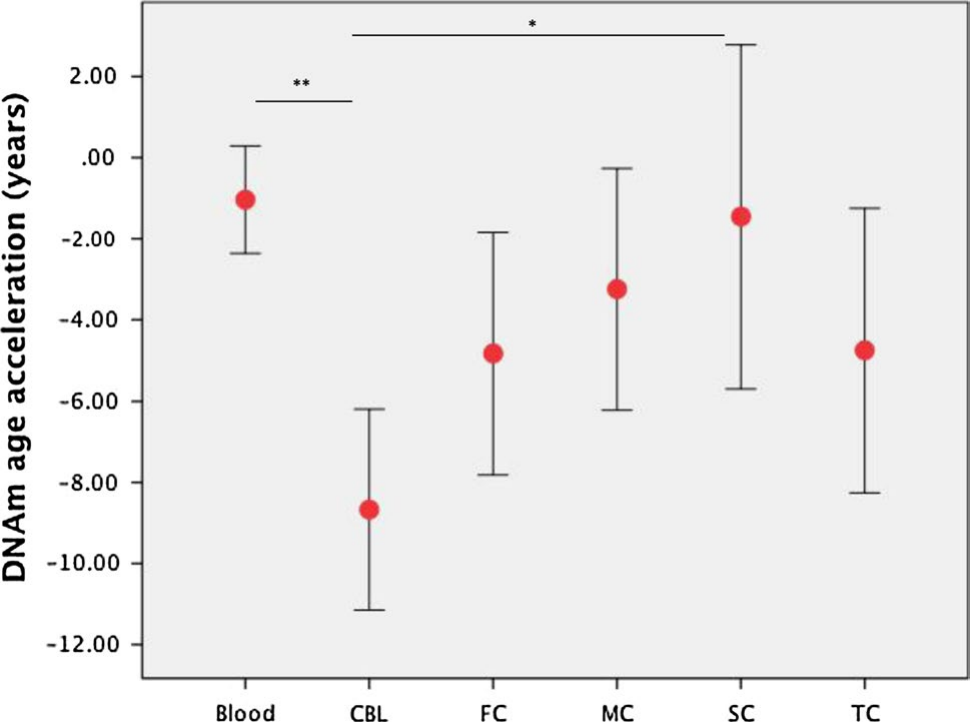
A comparison of DNAm age-acceleration across different tissues from *C9orf72* patients, including blood (*n* = 46), frontal cortex (FC, *n* = 10), cerebellum (CBL, *n* = 9), spinal cord (SC, *n* = 9), motor cortex (MC, *n* = 9), and temporal cortex (TC, *n* = 9). The blood DNAm age-acceleration was similar for all tissues except cerebellum (*p < 0.05, **p < 0.01)

We also assessed if the DNAm clock is synchronous across blood and CNS tissues of *C9orf72* patients (Fig. 6). The DNAm age-acceleration was similar between blood and CNS tissues (*p* > 0.05), except for cerebellum (*p* < 0.01), which has significantly lower DNAm age-acceleration compared to the other tissues: by 7.6 years vs. blood (*p* < 0.01), and 7.2 years vs. spinal cord (*p* = 0.02) (Fig. 6). Except cerebellum, the variation in DNAm age-acceleration among different tissues of the same individuals (*n* = 4) is small (standard deviation is 2–3 years for each case) (Table S5).

## Discussion

Our DNA methylation analysis of blood DNA of *C9orf72* patients revealed that acceleration of the aging process is significantly correlated with a more severe disease with a shorter disease duration and earlier age of onset. A similar trend was detected for the affected members of an extended *C9orf72* family, and a pilot study of CNS tissues of autopsy *C9orf72* cases (spinal cord, temporal and frontal cortex). Previous studies have reported accelerated DNAm aging in patients with Parkinson’s disease (using blood DNA) [14] and Huntington’s disease (using brain DNA) [12]. Also, DNAm age-acceleration was found to be correlated with the degree of Alzheimer’s disease related neuropathology, as well as cognitive and memory functions [16]. Current results further suggest the involvement of epigenetic aging in neurodegenerative diseases.

Importantly, DNAm age-acceleration was similar between blood and CNS tissues, except for cerebellum that ages more slowly. It is in agreement with a previous study of DNAm age in multiple tissues of centenarians, which revealed that the cerebellum is ~15 years younger than other tissues [13]. Of interest, the cerebellum in *C9orf72* cases is free from TDP-43 inclusions and neurodegeneration [20]; despite having a heavily misregulated transcriptome [23] and a high load of dipeptide repeat proteins (translated from the repeat expansion), which has been suggested to be toxic in some cell/animal models [19].

Since aging is the strongest risk factor for neurodegenerative diseases, differences in the aging process may contribute to the high heterogeneity observed in *C9orf72* patients [5, 21]. However, it is not clear if epigenetic factors react to aging or cause aging. The fact that DNAm age predicts chronological age more accurately in young vs. elderly individuals [10] suggests that environmental factors (e.g., smoking [15], diet and lifestyle [17]) interact with the genome during an individual’s entire life time and gradually modify DNA methylation status. For instance, in a pair of MZ ALS-discordant *C9orf72* twins, we observed increased DNAm age-acceleration in the affected twin compared to the asymptomatic twin [38], which is consistent with the current findings. Among the 353 CpGs contributing to DNAm age (hyper- or hypo-methylated with age), none showed a large (>10%) change in DNA methylation level between the twins [38], which suggests that the effect of DNA methylation on the aging process is driven by a set of CpGs with small effects rather than a single CpG with a major effect. Indeed, the current genome-wide methylation study of blood DNA from *C9orf72* carriers did not reveal any CpG with a methylation level significantly associated with disease duration or age of onset. In the future, a larger sample size would provide more power to detect the small effects of CpG methylation on variable disease presentation. Furthermore, a large *C9orf72* cohort would allow for the comparison of DNAm age-acceleration in ALS vs. FTD (not feasible in our study due to the limited number of FTD samples).

It would be important to assess more *C9orf72* families and CNS tissues to validate the correlation between DNAm age-acceleration, age of onset and disease duration, because the current study is limited to a small number of CNS tissues and only one *C9orf72* family. Furthermore, the question of whether the increased DNAm age-acceleration modifies the disease onset or it is a consequence of neurodegeneration is currently unclear. It would also be important to conduct a longitudinal analysis of DNAm age in *C9orf72* cases before and after disease onset. Of note, our previous longitudinal analysis of DNAm age in a pair of *C9orf72* identical twins discordant for ALS found that during a period of 4 years the affected twin had more stable DNAm age than the asymptomatic twin [38], suggesting that DNAm age might increase more rapidly prior to disease onset.

In summary, we report a significant association of DNAm age-acceleration with disease duration or age of onset in *C9orf72* carriers, suggesting that slowing the biological aging process may delay disease onset and progression. Importantly, blood DNAm age may be a useful biomarker, since it reflects the aging process in CNS. Indeed, blood DNAm age-acceleration has been reported to predict mortality in later life [22]. Understanding the links between aging and the clinical heterogeneity in *C9orf72* carriers may provide a clue for designing novel therapeutics, aiming to modulate DNAm age-acceleration to slow down disease progression or delay onset. Since ALS is a very severe disorder, with an average duration of only 2–5 years, even a small advancement in reducing its severity would be important for patients.

## Electronic supplementary material

Below is the link to the electronic supplementary material.

Supplementary material 1 (DOCX 36 kb)
