# Supplementary material for: DNA methylation age-acceleration is associated with disease duration and age at onset in C9orf72 patients
Source: Acta Neuropathol. 2017 Apr 24;134(2):271–9. doi: 10.1007/s00401-017-1713-y (PMC5508035; doi:10.1007/s00401-017-1713-y)
Supplement: Supplementary file 1 — Supplementary material 1 (DOCX 36 kb) [file 401_2017_1713_MOESM1_ESM.docx]

**Electronic supplementary materials**

**Table S1.** Genotyping results of *TMEM106B* (rs1990622, rs3153615) and *ATXN2* CAG repeats. MAF represents minor allele frequency.

| **Sample No.** | ***TMEM106B* rs1990622** | ***TMEM106B* rs3153615** | ***ATXN2* CAG repeats** | **Disease** |
| --- | --- | --- | --- | --- |
| 1 | AG | CG | 22/22 | ALS |
| 2 | AA | CC | 22/22 | ALS |
| 3 | AA | CC | 22/22 | ALS |
| 4 | AG | CC | 22/22 | ALS |
| 5 | AG | CG | 22/23 | ALS |
| 6 | AG | CG | 22/22 | ALS |
| 7 | AG | CG | 22/22 | ALS |
| 8 | GG | GG | 22/22 | ALS |
| 9 | AA | CC | 22/22 | ALS |
| 10 | AA | CC | 22/24 | ALS |
| 11 | AG | CG | 22/22 | ALS |
| 12 | AA | CC | 22/22 | ALS |
| 13 | GG | GG | 22/22 | ALS |
| 14 | AA | CC | 22/22 | ALS |
| 15 | AG | CG | 22/23 | ALS |
| 16 | AG | CG | 22/22 | ALS |
| 17 | AG | CG | 22/22 | ALS |
| 18 | AG | CG | 21/22 | ALS |
| 19 | AA | CC | 22/22 | ALS |
| 20 | AA | CC | 22/22 | ALS |
| 21 | GG | GG | 22/22 | ALS |
| 22 | GG | GG | 22/22 | ALS |
| 23 | AG | CG | 22/22 | ALS |
| 24 | AG | CG | 22/22 | ALS |
| 25 | AA | CC | 22/22 | ALS |
| 26 | AA | CC | 22/22 | ALS |
| 27 | AA | CC | 22/22 | ALS |
| 28 | AA | CC | 22/22 | ALS |
| 29 | AA | CC | 22/22 | ALS |
| 30 | AA | CC | 22/23 | ALS |
| 31 | GG | GG | 22/22 | ALS |
| 32 | GG | GG | 22/22 | ALS-FTD |
| 33 | AA | CC | 22/22 | ALS-FTD |
| 34 | AG | CG | 22/22 | ALS-FTD |
| 35 | AG | CG | 22/22 | ALS-FTD |
| 36 | AA | CC | 22/22 | ALS-FTD |
| 37 | AG | CG | 22/22 | ALS-FTD |
| 38 | AA | CC | 22/23 | FTD |
| 39 | AA | CC | 22/22 | FTD |
| 40 | AA | CC | 22/22 | FTD |
| 41 | AG | CG | 22/22 | FTD |
| 42 | AG | CG | 22/26 | FTD |
| 43 | AA | CC | 22/23 | FTD |
| 44 | AG | CG | 22/22 | FTD |
| 45 | AG | CG | 22/22 | FTD |
| 46 | AG | CG | 22/22 | FTD |
| MAF (All diseases, n=46) | 0.34 | 0.34 |  |  |
| MAF (ALS, n=31) | 0.35 | 0.35 |  |  |
| MAF (ALS-FTD, n=6) | 0.42 | 0.42 |  |  |
| MAF (FTD, n=9) | 0.28 | 0.28 |  |  |
| MAF (1000 Genomes) | 0.387 | Not available |  |  |

**Table S2**. The top 20 nominally significant CpGs detected by the genome-wide blood DNA methylation study: the result of the association between the locus-by-locus methylation changes and age of onset of *C9orf72* patients (p-value<0.0001, but q-value>0.05).

| CpG site | p-value | q-value | Gene |
| --- | --- | --- | --- |
| cg15202102 | 1.39E-06 | 0.414317471 | *PRKCDBP* |
| cg19817488 | 2.66E-06 | 0.414317471 | *CHCHD3* |
| cg00009871 | 3.27E-06 | 0.414317471 | *LOC121952* |
| cg00573770 | 4.23E-06 | 0.414317471 | *ZEB2* |
| cg10221746 | 6.95E-06 | 0.414317471 |  |
| cg02228185 | 7.37E-06 | 0.414317471 | *ASPA* |
| cg13931228 | 8.42E-06 | 0.414317471 | *MPP6* |
| cg22289662 | 8.75E-06 | 0.414317471 | *CUTA* |
| cg26053346 | 9.23E-06 | 0.414317471 | *LRIG1* |
| cg22900415 | 1.09E-05 | 0.414317471 | *GJA3* |
| cg19535369 | 1.14E-05 | 0.414317471 | *ZRSR2* |
| cg21572722 | 1.26E-05 | 0.414317471 | *ELOVL2* |
| cg09581137 | 1.31E-05 | 0.414317471 | *RUNDC2A* |
| cg03950689 | 1.34E-05 | 0.414317471 | *JAKMIP3* |
| cg16764274 | 1.41E-05 | 0.414317471 | *SGPP2* |
| cg06479512 | 1.69E-05 | 0.438815619 | *PNLDC1* |
| cg25694915 | 1.69E-05 | 0.438815619 | *NOSTRIN* |

**Table S3**. The top 20 nominally significant CpGs detected by the genome-wide blood DNA methylation study: the result of the association between the locus-by-locus methylation changes and disease duration of *C9orf72* patients (p-value<0.0001, but q-value>0.05).

| CpG site | p-value | q-value | Gene |
| --- | --- | --- | --- |
| cg01725130 | 2.49E-07 | 0.068429408 | *RIN3* |
| cg07464125 | 4.52E-07 | 0.068429408 | *KCNMA1* |
| cg00287312 | 6.39E-07 | 0.068429408 | *PCDH8* |
| cg09788778 | 1.29E-06 | 0.068429408 | *ZBTB12* |
| cg06229674 | 1.31E-06 | 0.068429408 | *APOBEC3H* |
| cg13751113 | 1.38E-06 | 0.068429408 | *AMICA1* |
| cg15636859 | 1.85E-06 | 0.068429408 | *RBM38* |
| cg02827278 | 1.94E-06 | 0.068429408 | *ORAI1* |
| cg08553524 | 2.17E-06 | 0.068429408 |  |
| cg10779336 | 2.31E-06 | 0.068429408 | *TREML2P* |
| cg01915525 | 2.59E-06 | 0.068429408 | *EP400NL* |
| cg04233054 | 2.72E-06 | 0.068429408 | *EPHB2* |
| cg14093936 | 2.84E-06 | 0.068429408 | *SEMA7A* |
| cg04736625 | 2.97E-06 | 0.068429408 | *KLRB1* |
| cg25029657 | 3.02E-06 | 0.068429408 | *ACOT7* |
| cg01906741 | 3.83E-06 | 0.068429408 | *LOC441089* |
| cg21847347 | 4.01E-06 | 0.068429408 | *RAB37* |
| cg00095678 | 4.52E-06 | 0.068429408 | *TMEM136* |
| cg25015416 | 4.53E-06 | 0.068429408 | *CNP* |

**Table S4.** The adjusted coefficient (Beta) and p-values for each variable from the multivariate linear regression analysis assessing the association between DNAm age-acceleration, age of onset and disease duration by controlling some factors.

| **Dependent variable: Age of onset** |  |  |
| --- | --- | --- |
|  | **Beta** | **p-value** |
| **DNAm age acceleration** | -0.334 | 0.025 |
| **Disease phenotype** | -0.169 | 0.25 |
| **Gender** | 0.299 | 0.047 |
| ***TMEM106B* rs1990622** | 0.279 | 0.402 |
| ***TMEM106B* rs3173615** | 0.295 | 0.372 |
| ***C9orf72* 5'CpG methylation** | 0.01 | 0.944 |
| **Dependent variable: Disease duration** |  |  |
|  | **Beta** | **p-value** |
| **DNAm age acceleration** | -0.519 | 0.00046 |
| **Disease phenotype** | 0.461 | 0.002 |
| **Gender** | 0.122 | 0.409 |
| ***TMEM106B* rs1990622** | 0.323 | 0.192 |
| ***TMEM106B* rs3173615** | 0.384 | 0.122 |
| ***C9orf72* 5'CpG methylation** | -0.074 | 0.583 |
| **Age of onset** | -0.269 | 0.077 |

**Table S5**. DNAm age-acceleration (years) across different tissues, each of which was available for four *C9orf72* patients: blood, frontal cortex (FC), cerebellum (CBL), Spinal cord (SC), motor cortex (MC), and temporal cortex (TC). D.B represents date of blood collection; D.A represents date of autopsy.

| Subject | Phenotype | Blood | FC | SC | MC | TC | CBL | D.B | D.A |
| --- | --- | --- | --- | --- | --- | --- | --- | --- | --- |
| 8863 | ALS-FTD | -0.37 | 2.79 | -1.20 | 5.85 | -3.99 | -10.17 | 2010-07-15 | 2011-10-24 |
| 8548 | ALS-FTD | -2.77 | -5.45 | -0.04 | -4.20 | -2.48 | -9.34 | 2009-02-25 | 2009-03-15 |
| 9090 | ALS | -5.03 | -7.64 | -2.16 | -9.86 | -11.00 | -11.16 | 2011-02-25 | 2013-03-13 |
| 9548 | ALS | 2.97 | 0.02 | -0.34 | -2.12 | 2.15 | -6.10 | 2012-10-24 | 2014-06-13 |
